# Comments on “Bamboo ecosystem services in 25 years: a systematic literature review of trends, insights, and knowledge gaps”

**DOI:** 10.1007/s11356-025-36998-w

**Published:** 2025-09-24

**Authors:** Kyoo-Man Ha

**Affiliations:** Rabdan Academy, Dhafeer St, Abu Dhabi, UAE

 Yuan (2025) as a corresponding author contributed to publishing a review article such as “Bamboo ecosystem services in 25 years: a systematic literature review of trends, insights, and knowledge gaps. 10.1007/s11356-025-36650-7.” This research tried to thoroughly analyze bamboo forest management by reviewing 56 prior studies before proposing future research directions (Li et al. 2025). A recurrent theme was that while many earlier studies focused on analyzing both maintenance and regulation services within the ecosystem service categories, they did not equally investigate the issue of interactions among ecosystem services.

Bamboo’s distinct traits enable it to offer a multitude of ecosystem services. As such, bamboo releases about 35% more oxygen than other trees and sequesters a considerable amount of carbon dioxide. Its extensive root system improves water infiltration and stabilizes the soil. Because bamboo provides habitat for a variety of species, it is also essential for biodiversity conservation (Mirzaie et al. 2025; Shah et al. 2025). It is a rapidly growing, renewable resource that promotes the sustainable production of goods like paper, textiles, and lumber without the need for replanting. Bamboo farming connects socioeconomic growth with environmental sustainability.

Due to the frequency, severity, and complexity of disasters, disaster management has taken on a crucial role in the twenty-first century (Coppola 2015). Natural hazards like wildfires, earthquakes with tsunamis, and climate change are examples of these, as are man-made emergencies like pandemics, terrorism, and mental stresses. Human society is now more interdependent and vulnerable via globalization and environmental degradation, which intensifies the impact of disasters. As a result, disaster management has developed into an internationally integrated subject that is important for promoting economic recovery, planetary health, and fairness in addition to saving lives.

By improving research and practical features, environmental science has greatly aided (or adopted) disaster management. In general, this has facilitated disaster management to change to a systems-based, resilience-focused, and preventive strategies (Mitra and Shaw 2023). Using a variety of instruments, environmental science has improved hazard identification, climate-disaster links, and ecosystem-based disaster risk reduction in research. In real life, environmental science has helped with land use planning, early warning systems, and post-disaster ecosystem restoration. It contributes to global frameworks like the Sendai Framework by interacting with local communities.

The unique ability of bamboo to reduce environmental hazards will lead to a growing interest in and use of bamboo and its ecosystem services in disaster management (also known as interactions among ecosystem services) (Isukuru et al. 2023). Bamboo’s deep root systems, quick growth, and capacity to sequester carbon make it a useful tool for controlling floodplains, repairing degraded land, and reducing climate change—all of which are major issues in disaster-prone areas. Ecosystems based on bamboo also improve biodiversity and nature-based solutions, and they maximize bamboo shelters during disaster reconstruction. Bamboo and ecosystem services will continue to supplement community resilience with sustainability.

More than currently, environmental science will further investigate and integrate disaster management in a variety of ways (Jonathan et al. 2025). For example, by incorporating ecological expertise into engineering, humanities, and social sciences, researchers can evaluate the ways in which bamboo’s ecosystem services lower the probability of calamity. The adoption of bamboo-based solutions will be made possible by cooperative efforts involving three organizational sectors: governments, corporations, and non-governmental organizations. Additionally, all phases of the disaster management cycle—disaster prevention/mitigation, preparedness, response, and recovery—will be equally adopted by environmental science (Rohana et al. 2025).

There will be a significant decrease in human casualties, financial losses, and psychological strains if environmental science further research and practice disaster management (Mubeen et al. 2023). To elaborate, environmental science may essentially raise the level of local capacities against a variety of risks while overcoming inadequate finance, a lack of leadership, public unawareness, or other obstacles. By doing these, communities will undoubtedly see fewer fatalities or psychological impacts. Bamboo will also provide sustainable building materials and livelihood support. These modifications will also open up new avenues for research in sustainable land use, climate adaptation, and socio-ecological systems.

## Data Availability

Not applicable.

## References

[CR1] Coppola DP (2015) Introduction to international disaster management. Butterworth-Heinemann, Sydney. 10.1016/C2014-0-00128-1

[CR2] Isukuru EJ, Ogunkeyede AO, Adebayo AA, Uruejoma MF (2023) Potentials of bamboo and its ecological benefits in Nigeria. Adv Bamboo Sci 4:100032. 10.1016/j.bamboo.2023.100032

[CR3] Jonathan MP, Marmolejo-Rodriguez AJ, Lopez E, Torres KB (2025) Our planet and our future. Environ Sci Pollut Res 32:3069–3071. 10.1007/s11356-024-35792-4 10.1007/s11356-024-35792-4 39722104

[CR4] Li CE, Lee SY, Chen YY, Kuo SY, Yuan MH (2025) Bamboo ecosystem services in 25 years: a systematic literature review of trends, insights, and knowledge gaps. Environ Sci Pollut Res. 10.1007/s11356-025-36650-7 10.1007/s11356-025-36650-7 PMC12274241 40560312

[CR5] Mirzaie A, Brandao M, Zarrabi H (2025) Toward eco-friendly menstrual products: a comparative life cycle assessment of sanitary pads made from bamboo pulp vs. a conventional one. Environ Sci Pollut Res 32:9050–9067. 10.1007/s11356-025-36269-8 10.1007/s11356-025-36269-8 PMC11968503 40100500

[CR6] Mitra A, Shaw R (2023) Systemic risk from a disaster management perspective: a review of current research. Environ Sci Policy 140:122–133. 10.1016/j.envsci.2022.11.022

[CR7] Mubeen M, Shabbir K, Hanif A, Ali M, Hussain S, Ahmad S (2023) Role of environmental science for disaster risk reduction in agriculture. In: Ahmed M, Ahmad S (eds) Disaster risk reduction in agriculture, 1st edn. Springer, Singapore, pp 131–145. 10.1007/978-981-99-1763-1_7

[CR8] Rohana R, Arni Y, Hakim L, Fitri EA (2025) Evolution of disaster preparedness studies: a bibliometric approach to exploring research trends and directions. Jamba J Disaster Risk Stud. 10.4102/jamba.v17i1.1800 10.4102/jamba.v17i1.1800 PMC11966665 40182532

[CR9] Shah AM, Ma C, Liu G, Liu Y, Shahbaz Z, Chen Q, Liu S (2025) Evaluating ecosystem services and disservices of bamboo forest using the emergy-based method. J Clean Prod 501:145092. 10.1016/j.jclepro.2025.145092

